# The Effect of Priming with Photographs of Environmental Settings on Walking Speed in an Outdoor Environment

**DOI:** 10.3389/fpsyg.2017.00073

**Published:** 2017-01-26

**Authors:** Marek Franěk, Lukáš Režný

**Affiliations:** Faculty of Informatics and Management, University of Hradec KrálovéHradec Králové, Czechia

**Keywords:** priming, walking speed, urban walking, viewing nature, approach-avoidance behavior, natural environment, positive affect

## Abstract

This study examined the effect of priming with photographs of various environmental settings on the speed of a subsequent outdoor walk in an urban environment. Either photographs of urban greenery, conifer forests, or shopping malls were presented or no prime was employed. Three experiments were conducted (*N* = 126, *N* = 88, and *N* = 121). After being exposed to the priming or no-priming conditions, the participants were asked to walk along an urban route 1.9 km long with vegetation and mature trees (Experiment 1, Experiment 3) or along a route in a modern suburb (Experiment 2). In accord with the concept of approach-avoidance behavior, it was expected that priming with photographs congruent with the environmental setting of the walking route would result in slower walking speed. Conversely, priming with photographs incongruent with the environmental setting should result in faster walking speed. The results showed that priming with the photographs with vegetation caused a decrease in overall walking speed on the route relative to other experimental conditions. However, priming with incongruent primes did not lead to a significant increase in walking speed. In all experimental conditions, the slowest walking speed was found in sections with the highest natural character. The results are explained in terms of congruency between the prime and the environment, as well as by the positive psychological effects of viewing nature.

## Introduction

There is considerable evidence suggesting that individuals have positive reactions to viewing nature. Several decades ago, Ulrich’s “Stress Recovery Theory” stated that viewing natural scenes has a positive effect on emotional and physiological states, tends to reduce stress and has a positive effect on individuals’ self-rated emotional state (e.g., Ulrich, 1981, 1986). Kaplan’s “Attention Restoration Theory” suggested that the effect of viewing nature restores fatigue from directed attention (Kaplan and Kaplan, 1989). In their meta-analysis, McMahan and Estes (2015) reported that exposure to natural environments is associated with a moderate increase in positive affect and a smaller decrease in negative affect relative to comparison conditions. Moreover, a number of studies (e.g., Stamps, 1990; Ulrich et al., 1991, 2003; Hartig et al., 1999; van den Berg et al., 2003; de Kort et al., 2006; Kweon et al., 2008; Valtchanov et al., 2010; Brown et al., 2013; Jiang et al., 2014; Felnhofer et al., 2015; Lottrup et al., 2015) have highlighted the benefits of exposing individuals to surrogate nature (photographs, slides, paintings, window views, videos, and virtual computer-generated nature scenes), which results in restorative effects, such as increased positive affect, decreased negative affect, and decreased stress.

Coherently, research also has shown the psychological benefits of walking outdoors in nature versus walking in urban settings (Hartig et al., 1991, 2003; Berman et al., 2008). Duvall (2011) investigated the role of cognitive engagement during urban walks. Strategies for cognitive engagement may utilize conscious thinking about the environment in which an individual is walking. Specifically, participants received a list of awareness plans designed to influence how they engaged in and interacted with the physical environment (e.g., “Focus on sounds. If the area is quiet, listen to the silence. If the area is full of sounds, focus on each one and notice how they differ”). The intention was to engage and involve the participants during their actual walking routine. It was found that the cognitive engagement with an outdoor environment helped participants to achieve significant improvements in multiple dimensions of psychological well-being. This technique is in some respect close to ecopsychological approaches (e.g., Roszak et al., 1995; Cohen, 2003; Voigt et al., 2007). Such approaches utilize techniques of working with all senses to obtain deep perception and understanding of the surrounding natural environment.

Taken together, we designed the present study from the findings of Duvall’s (2011) study and the experiences from ecopsychological techniques. In accordance with these findings, the idea was to expose participants in an urban “green” environment to certain stimuli prior to their walk, which would lead to a higher level of engagement and involvement in the environment. To address this question, the present study explored whether the technique of visual semantic priming can be used to strengthen cognitive engagement during a walk in an urban “green” environment. Specifically, the question here is whether exposure to series of photographs of urban nature (i.e., trees, grass, or alleys) prior to an outdoor walk in an urban “green” environment may enhance cognitive engagement with the outdoor walking environment. In contrast, with the previously mentioned approaches, priming with photographs of vegetation would represent more gentle stimulation than verbally expressed intention to attend to the surrounding natural features of the environment. Moreover, whereas previous studies analyzed mostly reported subjective experiences, we assessed direct behavioral responses, namely walking speed. In this regard, walking speed can reflect an individual’s emotional state and level of stress (e.g., Montepare et al., 1987; De Meijer, 1989; Crane and Gross, 2007; Hicheur et al., 2013), as well as interest in a particular environmental setting (see below).

Interestingly, in our previous research (Franěk, 2013; Franěk and Režný, 2014), we showed that participants’ walking speed during a short urban walk fluctuated as a response to the surrounding environment. They tended to walk slower in areas with less traffic, noise, and movement by people than in areas without greenery and with a higher level of traffic, noise, and movement by other people. Further analyses showed that participants felt more positive emotions in the locations where they spontaneously slowed down, and they were more likely in this environment and expressed a desire to affiliate with the environment in contrast with the locations where they spontaneously speeded up. This finding is in line with the *approach-avoidance behavior* described by Mehrabian and Russell (1974). Mehrabian-Russell’s theory posits that individual responses to an environment result from two different forms of behaviors: the individual is either trying to establish contact with the environment and remain inside it (approach behavior), or he/she is trying to avoid it and move away (avoidance behavior). The consequent reactions consist of various forms of behavior – physical movement heading into the environment (or outside it), a level of attention given to the features of the environment, exploration of the environment, communication with others in the environment, or an attempt to perform any kind of activity there. This theory has been specifically tested in consumer research (e.g., Bitner, 1992; Donovan et al., 1994; Wirtz et al., 2000; Jang and Namkung, 2009). Our previous investigation of urban walking (Franěk, 2013) showed that Mehrabian-Russell’s theory can also be used to explain affective reactions to outdoor urban environments. Specifically, approach behavior was observed as a response to urban greenery and was associated with slower walking speed, whereas avoidance behavior appeared in locations without greenery and with traffic and was associated with faster speed.

The technique of priming refers to the experimental paradigm in which a target stimulus is processed in the context of a stimulus previously encountered. The priming paradigm has been employed in various areas, such as research on memory (e.g., Tulving and Schacter, 1990), visual perception (for a review, see Kristjánsson and Campana, 2010), speech perception (e.g., Pisoni et al., 1985; Church and Fisher, 1998; Slowiaczek et al., 2000), music perception (Bharucha and Stoeckig, 1987), and other areas. Moreover, priming has been successfully used in studies on more complex forms of social behavior (for a review, see Bargh, 2006). Such research has shown that priming may affect conformity (Epley and Gilovich, 1999), politeness (Bargh et al., 1996), political beliefs (Carter et al., 2011), helping behavior (Garcia et al., 2002), social self-confidence (Steele and Aronson, 1995) and prosocial behavior (Shariff et al., 2016).

In numerous priming studies, the speed of a subsequent response has also been examined. For instance, a widely investigated approach in priming research is to record the speed, at which participants classify a target letter string after being exposed to a priming word (e.g., Meyer and Schvaneveldt, 1971). In a substantially different domain of research on behavior, it has been shown that priming can influence speed of movement in various sports activities (e.g., Follenfant et al., 2005; Ashford and Jackson, 2010). However, the effect of priming on walking speed has been examined by only a few studies. These studies have employed mechanisms of higher-level priming where indirect priming activated a concept (e.g., older age) that is in some way associated with an image of speed, or activated an image of animals that were characterized by a certain speed of movement. The activation of a concept or an image influenced participants’ subsequent behavior where participants assimilated behavior related to the primed concept or image.

Bargh et al. (1996) used a scrambled sentence task to prime the concept of “being old” and measured walking speed after leaving the laboratory. The participants had a slower walking speed in the elderly priming condition than participants in the neutral priming condition. Aarts and Dijksterhuis (2002) primed participants with names of fast or slow animals. Next, the participants were asked to walk to another location to receive instructions for a task. Participants primed with examples of slow animals walked slower than participants primed with examples of fast animals. Finally, Cesario et al. (2006) recorded the time it took subjects to leave the experiment room. They found that participants in the elderly prime condition had the slowest mean exit time, whereas participants had the fastest mean exit time in the youth prime condition.

The present study was designed to employ the mechanism of indirect priming to influence the speed of a subsequent walk. In contrast to prior research, our study attempted to combine a priming technique with current research from environmental psychology. We explored an association between exposure to a photograph of a specific outdoor scene (a technique widely used in environmental psychology) and the speed of walking in an outdoor environment. More specifically, the intention was to expose participants to photographs congruent with the environmental features of the outdoor route with vegetation (i.e., grass or trees) or photographs incongruent with the outdoor route (i.e., shopping malls) as primes. We assume that priming with two different types of environmental settings should have different effects on walking speed. Given that priming makes the mental representation of certain information more accessible (e.g., Loersch and Payne, 2011; Janiszewski and Wyer, 2014), we assumed that priming with photographs containing an urban setting with natural features will induce cognitive engagement and involvement during the walk through an urban route with greenery, which results in the approach behavior associated with slower walking speed. The effects of incongruent primes would be based on the contrast effect of a prime (Martin, 1986). The contrast effect in priming was frequently observed in social psychology experiments (for review, see Dijksterhuis and Bargh, 2001). The contrast effect appears if individuals perceive a primed concept to be inapplicable. Thus, the activated information influences interpretation or judgments in the opposite direction. It may be supposed that the contrast effect of an incongruent prime would result in avoidance behavior associated with a faster walking speed.

Despite the fact that many studies have shown that people prefer natural settings over urban ones, more recent investigations also analyzed the restorative potential of various outdoor and indoor urban settings. Recently, Staats et al. (2016) compared the restorative potential of various urban settings including shopping malls. As predicted, a park setting was most preferred, but malls and cafes also had some level of preference namely in a low attentional fatigue condition. Although there is no reason to expect gender differences in reactions to photographs with natural scenes, men and women differed in their reaction to photographs of shopping malls. Women rated the shopping malls more positively than did the men. The popular belief that men do not such as shopping and most women do such as shopping has been supported by several studies (e.g., Kruger and Byker, 2009; Ellis et al., 2012). Thus, liking of the stimuli and possible gender differences should also be controlled.

Any experiment conducted in a naturalistic setting can be influenced by various external variables. Clearly, it may be difficult to control for some of these variables, such as sudden changes in weather conditions and fluctuations in the intensity of city traffic. In previous investigations (Franěk, 2013; Franěk et al., 2014), we have addressed the effect of another external variables, such as physical features of the environment. Because our previous study (Franěk and Režný, 2014) was conducted in the same urban route as the route used in the current experiments, we are acquainted with the typical patterns of fluctuation in walking speed within this specific route and with their association with the environmental layout.

In sum, the purpose of the present study was to examine the effect of priming with photographs of certain types of outdoor environments on the subsequent speed of walking. The objective was to compare the effects of two types of visual primes: (1) photographs with vegetation that are congruent with the urban route with natural features, in which participants will consequently walk and (2) photographs without vegetation incongruent with this environment. The question was whether these different kinds of priming would influence the subsequent speed of walking.

## Experiment 1

The purpose of Experiment 1 was to study the effect of priming with photographs of urban greenery and photographs of shopping malls on participants’ walking speed along an urban route with greenery, trees, and alleys. The primes were either congruent (photographs of urban greenery) or incongruent (shopping malls) with the environmental features of the walking route.

### Method

#### Ethics Statement

Ethical approval was obtained from the Department of Management at the University of Hradec Králové for the experiment. The participants provided written informed consent in which they declared that they were voluntarily participating in the experiment and that they were informed about the experimental task, the procedures, and the apparatus used. They agreed that video recordings of their actions were made for scientific purposes only.

#### Participants

One hundred twenty-six undergraduates participated in the study. The students were young adults aged from 18 to 24 years (*M* age = 20.5 years, *SD* = 1.21), with 60 men and 66 women. They were enrolled in various psychology courses, but they were students of informatics, financial management, and tourism. They were compensated by partial course credit.

#### Walking Route

The walking route was a circuit through several alleys and streets with mature trees, gardens, and small houses in the central area of Hradec Králové. This city is located in the northeastern part of the Czech Republic and has approximately 100,000 inhabitants. First, the participants went from the university building to the starting point of the route, which was located approximately 300 m from the building. The circuit from the starting point to the final point was 1.9 km long. To compare walking speed in different locations of the route, we chose eight sections to measure walking speed (see **Table 1**). The sections were selected to provide a direct route and to avoid the ability to cross an intersection or similar obstacles. The sections were identical to the sections utilized in our previous study (Franěk and Režný, 2014).

**Table 1:** Walking route in Experiment 1.

Section	Length (m)	Environmental layout	Street
1	60	Grass, trees, traffic	*Brno Street – Technical High School*
2	55	Grass, trees, traffic	*Brno Street – Business High School*
3	100	Grass, trees, traffic	*Brno Street – Business Center*
4	100	Grass, trees, traffic	*Brno Street – Botanical Garden*
5	60	Dense oak alley, no traffic	*Brno Street – Malšovická Street*
6	75	Dense oak alley, no traffic	*Flošna – tree alley*
7	90	Dense oak alley, no traffic	*Flošna – parking*
8	80	Family houses, trees, traffic	*Nezvalova street*


*The description of particular sections, where walking speed was measured. For more details of the route, see https://maps.google.com, location: Hradec Kralove, Czech Republic, *nabrezi u Privozu*.*

#### Priming Material

Priming was conducted with a set of eight photographs, with either scenes of trees (henceforth “priming with trees”) or scenes of shopping malls (henceforth “priming with malls”). The photographs of the trees were taken in the city of Hradec Králové in alleys or in municipal parks, but they were not identical to the scenes of the walking route. The photographs of shopping malls were taken in various cities. These photographs showed both outdoor and indoor scenes (see Supplementary Material). The participants were asked to observe each photograph separately and to rate their level of liking of each image by using a scale ranging from 1 to 5. Viewing times and times for photographs ratings were not limited. For the rating, they used a paper sheet, which contained the rating scale. The task to rate the photographs would ensure that photographs will be carefully perceived by participants.

A between-subject design was employed. Forty-four participants (21 males, 23 females) were primed with trees; 42 participants, with shopping malls (20 males, 22 females). The no-priming group, which was not exposed to any priming, consisted of 40 participants (19 males, 21 females). The whole course of the 3 days experiment was divided into 1-h blocks. The participants registered to a specific block on your own terms. Subsequently, they were assigned to a particular experimental condition. The groups were balanced for participants’ study programs and gender.

#### Procedure

The map showing the walking route was mailed to the participants 1 week before participation. Upon arrival to the experiment room, the participants were asked to sign an informed consent form. Then, they read the instruction describing the walking task and looked at the route on a map. Afterward, a research assistant accompanied the participant to another room. Here, before their walk, the participants first filled out a *Positive and Negative Affect Schedule* (PANAS) questionnaire to measure their actual mood. Then, they were exposed to a priming task (trees or malls), or they were asked only to wait for the start of the walking task (no-priming control group). The research assistant carrying out the priming task was not familiar with the predicted effect of priming. Filling the PANAS questionnaire took approximately 3 min, and the priming task (or waiting in the no-priming condition) including the instruction lasted approximately 8–9 min.

After the participants complete the tasks, the research assistant accompanied the participants back to the experiment room. The time interval between the end of the priming procedure and the start of the walk was between 5 and 6 min. In the experiment room, the participants received a small video camera on a belt around their waist and were asked to go to the starting point of the route. At this point, they were contacted by another research assistant, who showed them the direction of the walk. When a participant finished the entire route, he/she returned to the experiment room and completed a post-walk PANAS questionnaire. The participants walked around the route individually, and the route was marked by noticeable orange arrows painted on the surface of a sidewalk to make orientation easier.

The study was conducted in 2013 on three workdays: October 29th (only during the afternoon), October 30th (during the whole day), and October 31st (during the whole day). The afternoon of October 29th was cloudy and slightly rainy, and the temperature was between 12 and 13°C. On the morning of October 30th, it was cloudy, and the temperature was between 8 and 9°C. During the afternoon, it was partly cloudy, and the temperature was between 12 and 13°C. On the morning of October 31st, there was sunshine, and the temperature was between 6 and 8°C. In the afternoon, it was sunny, and the temperature was between 12 and 13°C. The grass was still green, and deciduous trees were yellowed and partly bare. Because it was not possible to predict weather and actual atmospheric conditions, we made in advance a time schedule with a list of participants divided into 1-h sessions consisted of 6–8 participants. The time schedule was balanced by gender and the conditions across day and time.

#### Measurement of Walking Speed

The subjects walked with a small video camera (i.e., a Sony Bloggie MHS-PM5K) on a belt around their waist (size 19 mm × 108 mm × 55 mm, weight 110 g). Through a fish eye lens, the environment, the subject’s feet, and the subject’s arms were captured. Each section of the route had its beginning and end clearly indicated by a line drawn with intense color on the sidewalk. An evaluator marked two frames of the video recording to create the beginning and end of the annotation for each particular track section. Each frame corresponded to a time when a participant entered or left the section. The evaluation procedure consisted of annotating the video recordings in the software *Elan*. Annotations comprised the name of the track section and were time aligned to the video recordings. Every single annotation represented the entire section of the track, so that the extent of time subjects spend there could be determined. This enabled us to calculate the average speed reached by the subjects in all sections. The video recordings were processed by a team of research assistants.

#### Mood Measure

Actual mood before and after the walk were registered by the Czech translation of the PANAS (Watson et al., 1988). The original scale consisted of 20 adjectives describing positive and negative affect. In the Czech translation (for which a back translation procedure was used), a number of adjectives were removed. Seven adjectives were used to describe positive affect (Cronbach’s alpha = 0.75–0.80), and another seven adjectives were used for negative affect (Cronbach’s alpha = 0.76–0.79). For the original English scale, Cronbach’s alpha = 0.88 for positive affect and Cronbach’s alpha = 0.87 for negative affect (Watson et al., 1988).

#### Data Analysis

Both the duration of the walk and the walking speed in specific sections of the route were analyzed. The duration of the walk was measured from the beginning of section 1 to the end of section 8, including parts of the route between the sections where walking speed was not possible to measure. A two-way analysis of variance (ANOVA) was conducted to assess the effects of the experimental conditions (priming/no-priming conditions) and gender on the duration of the walk. Next, average walking speeds in specific sections of the route were calculated. To analyze the effects of both priming and the environmental properties of the route, a mixed ANOVA was conducted to assess the effects of the experimental conditions (priming/no-priming), gender, and the section of the route on the walking speeds in the specific sections. Because some sections on the walking route had similar environmental features and because participants walked in them at roughly same walking speed, we joined similar sections into four groups (i.e., sections 1 + 2 (group 1), sections 3 + 4 (group 2), sections 5 + 6 + 7 (group 3), and section 8 (group 4) in order to give the analyses more power to detect a significant interaction. The score for each group was the mean across included sections. Further, differences between reported mood before and after the walk were compared by using *t*-tests for independent samples. Differences between the ratings of photographs used as primes were compared by using *t*-test for independent samples. Finally, correlations between the duration of the walk, walking speeds in specific sections and the rating of photographs were calculated to assess, whether the level of liking of the photographs influences the walking speed. Statistical analyses were conducted using *Statistica 12*.

### Results

#### Duration of the Walk

The average duration of the walk for participants primed with trees from the beginning of section 1 to the end of section 8 was 657.34 s (*SD* = 45.68), while the average duration of the walk for participants primed with malls was 629.31 s (*SD* = 41.96). The average duration of the walk for participants in the no-priming group was 637.56 s (*SD* = 51.05). A two-way ANOVA showed a significant effect of the priming condition on the average duration of the walk (*F*_2,110_ = 3.78, *p* < 0.05, η^2^ = 0.06). The gender had no a significant effect (*F*_1,10_ = 1.05, *p* = 0.31). A *post hoc* Tukey test revealed that the participants primed with trees walked on the route significantly slower than the participants primed with malls. The effect size was medium (Cohen’s *d* = 0.64). However, the no-priming condition did not significantly differ from the priming with trees condition (Cohen’s *d* = 0.41), nor from the priming with malls condition (Cohen’s *d* = 0.17).

#### Walking Speed

**FIGURE 1:**
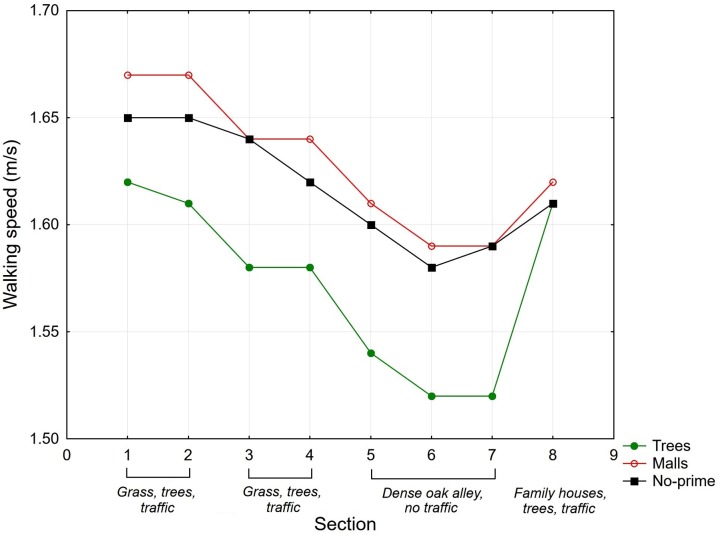
The average walking speeds (m/s) in eight sections of the route for the priming with trees, priming with malls, and no-priming conditions in Experiment 1.

The average walking speeds in particular sections are shown in **Figure 1** and **Table 2**. A mixed ANOVA was conducted to access the effects of the priming condition, gender of the participants, and environmental characteristics of the route on walking speed. The condition (priming with trees vs. priming with malls vs. no-priming condition) and participants’ gender were chosen as the categorical predictors, the section of the route (level 1 = sections 1 + 2, level 2 = sections 3 + 4, level 3 = sections 5 + 6 + 7, level 4 = section 8) was chosen as the within-subject (repeat measures) factor, and the speed of walking was used as the dependent variable. The ANOVA indicated a statistically significant within-subjects main effect of section walked (*F*_3,291_ = 61.63, *p* < 0.001, η^2^ = 0.39) and statistically significant between-subjects main effect of the priming condition (*F*_2,97_) = 3.06, *p* < 0.05, η^2^ = 0.06). Gender did not have a significant effect (*F*_1,97_ = 2.86, *p* = 0.09, η^2^ = 0.03), and there were no significant interaction between the priming condition and section walked (*F*_6,291_ = 1.46, *p* = 0.19, η^2^ = 0.03).

**Table 2:** The average walking speeds (m/s) in specific sections of the route for the two experimental conditions (trees and malls) and no-priming condition in Experiment 1.

Section	Priming “trees”		Priming “malls”		No-prime condition	
			
	Mean	*SD*	Mean	*SD*	Mean	*SD*
1	1.62	0.12	1.67	0.12	1.65	0.14
2	1.61	0.12	1.67	0.12	1.65	0.13
3	1.58	0.13	1.64	0.11	1.64	0.13
4	1.58	0.11	1.64	0.12	1.62	0.14
5	1.54	0.14	1.61	0.12	1.60	0.14
6	1.52	0.12	1.59	0.12	1.58	0.15
7	1.52	0.13	1.59	0.12	1.59	0.15
8	1.61	0.17	1.62	0.12	1.61	0.15


Walking speed was slower when participants were primed with trees than when they were primed with malls or not subjected to priming. A *post hoc* Tukey test showed significant differences in walking speed between the priming with trees condition and the priming with malls condition, as well as between the priming with trees condition and the no-priming condition. The priming with malls condition did not significantly differ from the no-priming condition. Walking speed differed between the groups of sections, where sections 5 + 6 + 7 with more natural characteristics were associated with the lowest walking speed. A *post hoc* Tukey test showed statistically non-significant differences in walking speed between sections 3 + 4 and section 8.The differences between walking speeds in the other sections were statistically significant.

#### Changes in Mood after the Walk

The data revealed (**Table 3**) that in all three conditions, reported positive affect after the walk did not change, while negative affect significantly declined in all three conditions.

**Table 3:** Scores for negative and positive mood of the participants before and after the walk for the two experimental (trees and malls) and no-priming condition in Experiment 1 and Experiment 2.

Condition	Positive mood						Negative mood					
		
	Before		After		*t*	*p*	Before		After		*t*	*p*
						
	Mean	*SD*	Mean	*SD*			Mean	*SD*	Mean	*SD*		
**Experiment 1**												
Priming “trees”	21.20	3.39	21.49	4.29	0.557	N.S.	10.44	2.85	8.81	2.23	2.952	*p* < 0.01
Priming “malls”	20.28	4.63	20.69	5.60	0.373	N.S.	10.57	2.57	9.33	2.78	2.010	*p* < 0.05
No-prime condition	20.00	4.40	21.18	4.32	1.257	N.S.	11.63	3.79	8.9	2.80	3.660	*p* < 0.001
**Experiment 2**												
Priming “trees”	30.85	7.12	30.59	8.31	0.146	N.S.	17.15	5.05	12.87	5.94	3.429	*p* < 0.001
Priming “malls”	31.49	5.85	31.89	6.66	0.255	N.S.	18.06	5.29	13.97	5.63	3.041	*p* < 0.01
No-prime condition	30.75	6.24	31.44	7.40	0.286	N.S.	16.38	3.83	12.56	2.78	3.223	*p* < 0.01


*In Experiment 1 the scale ranged from 1 to 5, in Experiment 2 the scale ranged from 1 to 7.*

#### Ratings of Photographs Used as Primes

The images with trees were liked more than photographs of shopping malls. *T*-tests for independent samples revealed significant differences between the average rating of trees and malls (*t* = 6.57, *p* < 0.001, Cohen’s *d* = 1.60). The effect size was large. There were no gender differences in ratings of the photographs. There were no any significant correlations between the duration of the walk and the rating of photographs (*r* values ranged from 0.24 to 0.25), nor any significant correlations between walking speeds in specific sections and the rating of photographs (*r* values ranged from 0.01 to 0.20).

### Discussion

The results showed that priming with photographs had a significant effect on walking speed. The participants exposed to priming with trees walked significantly more slowly than the participants primed with malls and the participants in the no-priming group. Although priming with malls resulted in slightly faster walking speed relative to the no-priming control condition, this difference was not significant. The results further showed that participants primed with trees consistently walked more slowly in almost all sections (except the last section) of the route than the participants assigned to the other conditions. The slowest walking speed was observed for section 3 (sections 5 + 6 + 7), which was located in a long, dense alley. A similar result was found in our previous study (Franěk and Režný, 2014). The decrease in walking speed in some sections of the route reflects the greater natural characteristics of the location, as found in the previously mentioned study.

We did not find any differences in post-walk affective states related to type of priming or to liking of photographs. The walk decreased negative affect regardless of the priming condition. This finding is congruent with the results of studies showing that short urban recreational walks (e.g., Ekkekakis et al., 2000; Johansson et al., 2011) and outdoor runs (e.g., Bodin and Hartig, 2003) were associated with shifts toward more positive affect.

In sum, the experiment did not convincingly answer the question regarding the mechanisms that might underlie the observed effect of the priming. Priming with photographs congruent with the environment of the walking route decreased participants’ walking speed, which may be explained in terms of approach behavior. Approach behavior means that an individual has increased interest in the environment, which leads to an attempt to explore the environment and potentially results in decreased walking speed. However, priming with photographs incongruent with the environment of the walking route did not lead to avoidance behavior because differences between the priming with malls condition and the no-priming condition were not statistically significant.

## Experiment 2

The goal of Experiment 2 was to study the effect of priming on walking speed in the environment of a walking route, which was not congruent with the primes. The same primes as in Experiment 1 (trees, malls, no priming) were used, but the walking route was located in a different urban environment in Hradec Králové that was visually incongruent with the primes. The environment of the walking route encompassed streets in a suburb with estates built between 1970s and 1980s with concrete apartment buildings. Part of the walking route was along a busy road, and another part was along a quiet street. Although grass and trees are present between the buildings, the environment substantially differed from the environment of the route in Experiment 1. Since we assume that the decrease in walking speed was due to congruence between the prime and the environment and consequent approach behavior, we predict that walking speed should not differ between all conditions.

### Method

#### Participants

Eighty-eight undergraduates participated in the study. The students were young adults between the ages of 18 and 23 years (*M* age = 21.0 years, *SD* = 1.02), with 43 men and 45 women. They were enrolled in various psychology courses, but they were students of informatics, financial management, and tourism. They were compensated by partial course credit. Sixty-four participants had already taken part in Experiment 1 (32 males, 32 females), while 24 participants (11 males, 13 females) participated in this experiment for the first time. Since the sample encompassed participants with different familiarity with the task, the data were also analyzed separately for both groups.

#### Walking Route

The walking route was a circuit through a modern suburb of the city of Hradec Králové. First, the participants went from the university building to the starting point of the route, which was located approximately 200 m from the building. From the starting point to the final point, the circuit was 1.2 km long. The time duration of the overall walk was measured from the beginning of section 1 to the end of section 8 (see **Table 4**).

**Table 4:** Walking route in Experiment 2.

Section	Length (m)	Environmental layout	Street
1	60	Houses, trees, traffic	*Hradecká street*
2	65	Houses, trees, traffic	*Hradecká street*
3	60	Houses, grass, heavy traffic	*Sokolská street*
4	45	Houses, grass, heavy traffic	*Sokolská street*
5	50	Houses, grass, trees, heavy traffic	*Sokolská street*
6	60	Houses, grass, heavy traffic	*Sokolská street*
7	70	Houses, grass, no traffic	*Akademika Heyrovského street*
8	50	Houses, grass, no traffic	*Akademika Heyrovského street*


*The description of particular sections, where the walking speed was measured. For more details of the route, see https://maps.google.com, location: Hradec Kralove, Czech Republic, *Akademika Heyrovskeho*.*

#### Priming Material

The priming material was identical to the priming material used in Experiment 1.

#### Procedure

The procedure was identical to the procedure employed in Experiment 1. Thirty-nine participants (20 males, 19 females) were primed with trees, and 33 participants were primed with shopping malls (14 males, 19 females). The no-priming group, which was not exposed to any priming, consisted of 16 participants (nine males, seven females). The participants, who already took part in Experiment 1, were exposed to a different type of priming than in the previous experiment or to no priming. Before their walk, the participants first filled out a PANAS questionnaire. When a participant finished the entire route, he/she returned to the experiment room and completed a post-walk PANAS questionnaire.

The study was conducted in 2014 during 2 weeks on four workdays: April 16th, April 17th, April 23rd, and April 24th. During the morning of April 16th, there was sunshine, and the temperature was 9°C. During the afternoon of April 16th, it was cloudy, and the temperature was 10°C. During the morning of April 17th, there was sunshine, and the temperature was between 6 and 10°C. On the afternoon of April 17th, there was sunshine, and the temperature was between 12 and 13°C. On the morning of April 23rd, it was cloudy, and the temperature was between 11 and 13°C and then decreased to 8°C. On the afternoon of April 23rd, it was partly cloudy, and the temperature was between 15 and 20°C. On the morning of April 24th, there was sunshine, and the temperature was between 12 and 18°C. During the afternoon of April 24th, it was sunny, and the temperature was between 20 and 22°C. The grass was already green, and the trees were light green with sparse foliage. The time schedule was balanced by gender and the conditions across day and time.

### Results

#### Duration of the Walk

The average duration of the walk of participants primed with trees measured from the beginning of section 1 to the end of section 8 was 635.45 s (*SD* = 45.41), while the average duration of the walk of participants primed with malls was 621.45 s (*SD* = 48.53). The average duration of the walk of participants in the no-priming group was 612.76 s (*SD* = 33.30). Although priming with trees resulted in a longer walking duration than priming with malls and no priming, a two-way ANOVA did not indicate a significant effect of condition on the average duration of the walk (*F*_2,81_ = 1.71, *p* = 0.19), nor statistically significant effect of gender (*F*_1,81_ = 0.56, *p* = 0.45).

In the next step, we analyzed the data for both groups of participants separately. First, we analyzed data of the participants who had already participated in Experiment 1. The average duration of the walk of participants primed with trees was 630.54 s (*SD* = 47.84), the average duration of the walk of participants primed with malls was 623.93 s (*SD* = 49.11), and the average duration of the walk of participants in the no-priming group was 623.01 s (*SD* = 30.49). A two-way ANOVA did not show a significant effect of condition on the duration of the walk (*F*_2,58_ = 0.17, *p* = 0.84), nor the effect of gender was significant (*F*_1,58_ = 0.01, *p* = 0.98).

Next, we analyzed data of the participants who did not take part in Experiment 1. The average duration of the walk of participants primed with trees was 647.51 s (*SD* = 38.14), the average duration of the walk of participants primed with malls was 613.80 s (*SD* = 49.20), and the average duration of the walk of participants in the no-priming group was 590.10 s (*SD* = 30.00). In contrast to the group of participants familiar with the experiment, a two-way ANOVA yielded a significant effect of condition on the average duration of the walk (*F*_2,17_ = 4.44, *p* < 0.05, η^2^ = 0.04). The effect of gender was not significant (*F*_1,17_ = 3.09, *p* = 0.10). A *post hoc* Tukey test revealed significant differences between the priming with trees condition and the no-priming condition, and the effect size was high (Cohen’s *d* = 1.66).

#### Walking Speed

**FIGURE 2:**
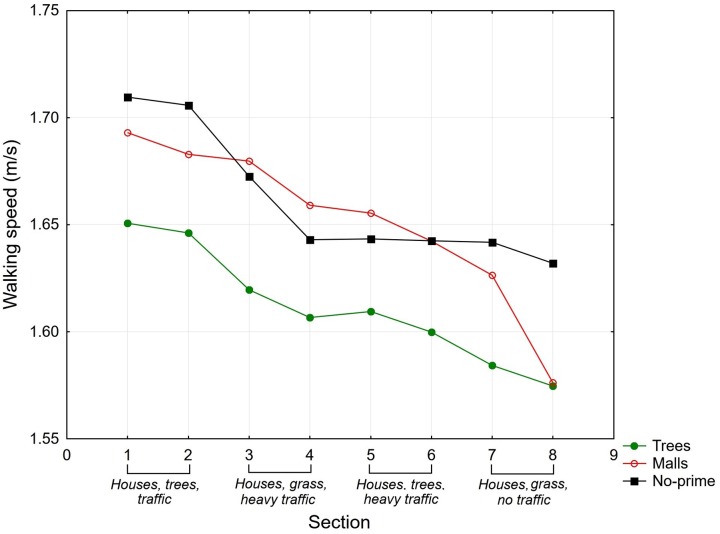
The average walking speeds (m/s) in eight sections of the route for the priming with trees, priming with malls, and no-priming conditions in Experiment 2.

The average walking speeds in particular sections are shown in **Figure 2** and **Table 5**. First, we conducted a mixed ANOVA on the data for all participants. The condition (priming with trees vs. priming with malls vs. no-priming condition) and participants’ gender were chosen as the categorical predictors, the section of the route (level 1 = sections 1 + 2, level 2 = sections 3 + 4, level 3 = sections 5 + 6, level 4 = sections 7 + 8) was chosen as the within-subject (repeat measures) factor, and the speed of walking was used as the dependent variable. The ANOVA showed a statistically significant within-subjects main effect of section walked (*F*_3,240_ = 29.92, *p* < 0.001, η^2^ = 0.27). However, the between-subjects main effect of both the priming condition (*F*_2,80_ = 1.41, *p* = 0.25) and gender (*F*_1,80_ = 0.38, *p* = 0.54) were not significant, nor were any of the interactions.

**Table 5:** The average walking speeds (m/s) in specific sections of the route for the two experimental conditions (trees and malls) and no-priming condition in Experiment 2.

Section	Priming “trees”		Priming “malls”		No-prime condition	
			
	Mean	*SD*	Mean	*SD*	Mean	*SD*
1	1.65 (1.61)	0.12 (0.09)	1.69 (1.69)	0.14 (0.15)	1.71 (1.76)	0.10 (0.10)
2	1.65 (1.60)	0.12 (0.07)	1.68 (1.68)	0.12 (0.13)	1.71 (1.76)	0.11 (0.13)
3	1.62 (1.57)	0.13 (0.08)	1.68 (1.69)	0.15 (0.14)	1.67 (1.74)	0.10 (0.12)
4	1.61 (1.58)	0.13 (0.08)	1.66 (1.67)	0.14 (0.16)	1.64 (1.71)	0.11 (0.10)
5	1.61 (1.59)	0.12 (0.10)	1.66 (1.67)	0.14 (0.17)	1.64 (1.72)	0.11 (0.10)
6	1.60 (1.58)	0.12 (0.11)	1.64 (1.66)	0.14 (0.17)	1.64 (1.70)	0.10 (0.09)
7	1.58 (1.56)	0.12 (0.11)	1.63 (1.66)	0.14 (0.17)	1.64 (1.72)	0.10 (0.07)
8	1.57 (1.56)	0.12 (0.11)	1.58 (1.62)	0.19 (0.13)	1.63 (1.70)	0.09 (0.08)


*The table separately shows data for the whole sample and for the first timers group (in parentheses).*

Next, we conducted an analysis on only the group of participants who did not take part in Experiment 1. The ANOVA showed a statistically significant between-subjects main effect of the priming condition (*F*_2,17_ = 4.65, *p* < 0.05, η^2^ = 0.36) and a significant between-subjects main effect of gender (*F*_2,17_ = 4.38, *p* < 0.05, η^2^ = 0.20). However, the within-subjects main effect of section walked was not significant, *F*_3,51_ = 1.93, *p* = 0.14), nor were any of the interactions. A *post hoc* Tukey test showed significant differences between the priming with trees condition and the no-priming condition and significant differences between males and females. The participants primed with trees walked significantly slower than the participants in the no-priming condition, and females walked slower than males.

#### Changes in Mood after the Walk

As in Experiment 1, the data revealed (**Table 3**) that in all three conditions, reported positive affect after the walk did not change, while negative affect significantly declined in all three conditions.

#### Rating of Photographs Used as the Primes

The images of trees were liked more than photographs of shopping malls. *T*-tests for independent samples revealed a significant difference in the average ratings between images with trees and those with malls (*t* = 10.52, *p* < 0.001). The effect size was large (Cohen’s *d* = 2.48). There were no gender differences in ratings of the photographs. There were no any significant correlations between the duration of the walk and the rating of photographs (*r* values ranged from 0.01 to 0.08), nor any significant correlations between walking speeds in specific sections and the rating of photographs (*r* values ranged from 0.01 to 0.19).

### Discussion

The results showed that priming with trees resulted in slower walking speed relative to priming with malls and no priming in an environment that was not congruent with the primes. However, it is worth commenting on the level of congruence/incongruence between the primes and the outdoor setting. Although the environment did not include large trees and alleys, there were still some low trees and greenery there.

As in Experiment 1, the walking speed on the route was also influenced by the features of the environment. The participants in both groups walked faster at the beginning of the route, which was close to a crossroad, as well as on a sidewalk located along a street with intense car traffic.

It should be noted that the effect of priming was significant only for the first time group. Recent evidence has accumulated in cognitive neuroscience (Dehaene et al., 2001, 2006) showing that two factors are necessary for semantic priming to occur – attention to the prime and stimulus saliency to the participants. Importantly, the participants who were familiar with the experiment devoted less attention and showed less interest in the priming because they were already familiar with the task from the previous experiment. However, the limitation of this finding is that the first time group was rather small.

## Experiment 3

The purpose of Experiment 3 was to compare the effects of priming with photographs of two different types of environments with trees and greenery. The experiment was conducted with the same walking route as Experiment 1. Two types of “green” environments were used as the primes. The first one, images of trees, had already been used in Experiment 1 and Experiment 2. These photographs were congruent with the environment of the walking route. The second type of “green” prime was photographs of conifer forests. These photographs were of conifer trees from submontane forests from hilly landscapes and were entirely incongruent with the environment of the urban walking route with deciduous tree. They were taken in submontane forests in north eastern parts of the Czech Republic. Because local people often visit these forests during their leisure activities, it was supposed that our participants were familiar with this type of outdoor environment and can recognize differences between these two “green” environments. The third type of prime was photographs of malls, which were used in both of the previous experiments. Because our assumption was that the decrease in walking speed was due to congruence between the prime and the environment and consequent approach behavior, we expected that the walking speed under priming with incongruent conifer forests would differ from walking speed under priming with trees congruent with the walking route.

In addition, we also looked for walking speed very early after the participants left the university building, outside the walking route. This area of interest was located close to modern concrete buildings and was not congruent with any of the types of priming. The purpose was to explore at what point the differences in walking speed between the particular experimental conditions appeared. Did they appear as soon as the participants left the building or they reached the first sections of the walking route as a response to the green environment of the route?

### Method

#### Participants

One hundred twenty-one undergraduates participated in the study. The students were young adults aged from 18 to 24 years (*M* age = 20.6 years, *SD* = 1.11), with 53 men and 68 women. They were enrolled in various psychology courses, but they were students of informatics, financial management, and tourism. They were compensated by partial course credit. No one took part in the previous experiments.

#### Walking Route

The walking route was identical to the route used in Experiment 1. Moreover, we added section 0, which was located outside the walking route close to the university building. Clearly, it was quite difficult to measure participants’ walking speed immediately after they left the experiment room or the university building, as the participants did not walk an identical or straight line in this area. The measurements of walking speed close to the university building were carried out only in a straight section located approximately 180 m from the entrance to the university building. This was a straight section leading along a sidewalk, which was 136 m long. This route was located on a street surrounded by a large modern concrete building and a building of another school.

#### Priming Material

Priming was conducted with a set of eight photographs, with scenes of trees, scenes of forests, or scenes of shopping malls. The scenes of trees and shopping malls were identical to the photographs used in Experiment 1 and Experiment 2. The scenes of forest were taken from various conifer forests in the northern parts of the Czech Republic and contained photos of conifers and paths leading through woods (see Supplementary Material).

#### Procedure

The procedure was modified to some extent. To assure an immediate effect of priming, the time between priming and the initiation of the walk was considerably shortened. In contrast to the previous experiments, the participants immediately started to walk after completing the priming. Given that significant difference between the priming with malls condition and the no-priming conditions were not observed in previous experiments, the no-priming condition was not employed for practical reasons (the number of experimental conditions and time limitation given by desire to conduct the experiment within 1 week). Because we did not find any differences in mood changes between the particular conditions in Experiment 1 and Experiment 2, we did not assess mood before and after the walk.

Upon arrival to the experiment room, the participants were asked to sign an informed consent form. Then, they were asked to read instructions concerning the walking task and to look at the route on a map. Afterward, the participants received a small video camera on a belt to be placed around their waist. Then, a research assistant accompanied the participants to another room, where they were primed with trees, forests, or malls. The room was located close to the entrance of the university building (approximately 10 m). When the priming was completed, the participants were asked to leave the room and to go to the starting point of the route, which was approximately 300 m from the building.

Forty-two participants (18 males, 24 females) were primed with trees, 40 participants were primed with forest (18 males, 22 females), and 39 participants were primed with shopping malls (17 males, 22 females). The study was conducted in 2014 on three workdays: October 21st (only during the afternoon), October 22nd (during the whole day), and October 23rd (during the whole day). During the afternoon of October 21st, it was cloudy, and the temperature was between 14 and 17°C. On October 22nd, it was cloudy, and the temperature was between 14 and 17°C. On October 23rd, it was cloudy or overcast, and the temperature was between 8 and 9°C. The grass was still green, and deciduous trees were yellowed and partly bare. The time schedule was balanced by gender and the conditions across day and time.

#### Measurement

The measurements were carried out in the same way as in the previous experiments.

### Results

#### Duration of the Walk

The average duration of the walk of participants primed with trees measured from the beginning of section 1 to the end of section 8 was 663.00 s (*SD* = 73.21). The average duration of the walk of participants primed with forests was 653.95 s (*SD* = 57.22), while the average duration of the walk of participants primed with malls was 618.46 s (*SD* = 69.60). A two-way ANOVA showed a significant effect of condition on the average duration of the walk (*F*_2,105_ = 6.49, *p* < 0.01, η^2^ = 0.11) and a significant effect of gender on the average duration of the walk (*F*_1,105_ = 7.64, *p* < 0.01, η^2^ = 0.07). The interaction between condition and gender was nearly significant (*F*_2,105_ = 3.00, *p* = 0.054, η^2^ = 0.05). A *post hoc* Tukey HSD test revealed that the participants primed with trees walked on the route significantly slower than the participants primed with malls. The effect size was medium (Cohen’s *d* = 0.634). Females walked slower than males. The interaction between condition and gender means that under all priming conditions the duration of the female’s walk was almost equal, while priming with trees and forests resulted in slower walking speed than priming with malls only in males.

#### Walking Speed

**FIGURE 3:**
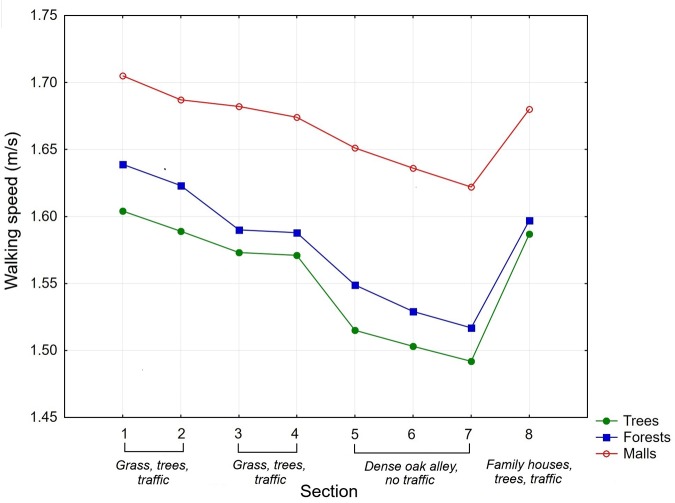
The average walking speeds (m/s) in eight sections of the route for the priming with trees, priming with forests, and priming with malls in Experiment 3.

The average walking speeds in particular sections are shown in **Figure 3** and **Table 6**. A mixed ANOVA was conducted to access the effects of the priming condition, gender of the participants, and environmental characteristics of the route on walking speed. Conditions (priming with trees vs. priming with forests vs. priming with malls) and participants’ gender were chosen as the categorical predictors, the section of the route (level 1 = sections 1 + 2, level 2 = sections 3 + 4, level 3 = sections 5 + 6 + 7, level 4 = section 8) was chosen as the within-subject (repeat measures) factor, and the speed of walking was used as the dependent variable. The ANOVA showed a statistically significant within-subjects main effect of section walked (*F*_3,312_ = 60.26, *p* < 0.001, η^2^ = 0.37) and a statistically significant between-subjects main effect of priming condition (*F*_2,104_ = 7.82, *p* < 0.001, η^2^ = 0.13) and gender (*F*_1,104_ = 9.84, *p* < 0.01, η^2^ = 0.09). There was also a significant interaction between condition and gender (*F*_2,104_ = 4.78, *p* < 0.01, η^2^ = 0.08). The walking speed between the groups of sections differed, where sections 5 + 6 + 7 with more natural characteristics were associated with the lowest walking speed. A *post hoc* Tukey test showed significant differences in walking speed between the priming with trees condition and the priming with malls condition, as well as between the priming with forests condition and the priming with malls condition. The participants primed with trees and forests walked more slowly than the participants primed with malls. In general, males walked significantly faster than females. The next finding was the significant interaction between condition and gender. In this regard, while priming with trees and forests resulted in slower walking speed than priming with malls for males, each priming condition resulted in almost equal walking speed in females.

**Table 6:** The average walking speeds (m/s) in specific sections of the route for the three experimental conditions (trees, forests, and malls) in Experiment 3.

Section	Priming “trees”		Priming “forests”		Priming “malls”	
			
	Mean	*SD*	Mean	*SD*	Mean	*SD*
1	1.60	0.16	1.64	0.14	1.71	0.18
2	1.59	0.17	1.62	0.14	1.69	0.19
3	1.57	0.18	1.59	0.13	1.68	0.19
4	1.57	0.18	1.59	0.14	1.67	0.18
5	1.52	0.17	1.55	0.17	1.65	0.19
6	1.50	0.18	1.52	0.16	1.64	0.19
7	1.50	0.17	1.52	0.15	1.62	0.18
8	1.59	0.16	1.60	0.13	1.68	0.17


Finally, we analyzed walking speed in section 0, which was located close to the university building. The average walking speed of participants primed with trees was 1.69 m/s (*SD* = 0.17), the average walking speed of participants primed with forests was 1.69 m/s (*SD* = 0.20), and the average walking speed of participants primed with malls was 1.82 m/s (*SD* = 0.22). Thus, the participants primed with trees and forests walked more slowly than the participants primed by malls already in the section of the route that was not visually congruent with any of the priming photographs. An ANOVA showed a significant effect of condition (*F*_2,86_ = 5.44, *p* < 0.01, η^2^ = 0.11) and a significant interaction between gender and condition (*F*_2,860_ = 4.27, *p* < 0.05, η^2^ = 0.09. A *post hoc* Tukey test showed significant differences in walking speed between priming with forests and priming with malls. Cohen’s *d* was 0.66.

#### Ratings of Photographs

In general, the images with trees and forests were liked more than photographs of shopping malls. *T*-tests for independent samples revealed a significant difference in average ratings between images of trees and images of malls (*t* = 5.70, *p* < 0.001, Cohen’s *d* = 1.29) and between images of forests and images of malls (*t* = 5.26, *p* < 0.001, Cohen’s *d* = 1.20). The effect sizes were large.

Although we did not find any significant effect of priming condition on walking speed for women, we looked for possible gender differences in evaluations of the photographs. Interestingly, in contrast with Experiment 1 and Experiment 2 the data showed (see **Table 7**) that women exposed to priming with malls on average rated the photographs of malls more highly than men (*t* = 2.19, *p* < 0.05, Cohen’s *d* = 0.26). There was only a difference in evaluations of two photographs taken outside malls that also showed cars, where males rated these photographs more highly than females (*t* = 2.03, *p* < 0.05, Cohen’s *d* = 0.64).

**Table 7:** Rating of the photographs of malls separately for male and female participants.

	Males		Females		
			
Photographs no.	Mean	*SD*	Mean	*SD*	Setting
1	2.81	1.05	2.29	0.85	Outdoor
2	2.38	1.09	3.05	1.12	Indoor
3	3.56	1.03	3.67	1.11	Indoor
4	3.63	1.09	4.00	1.10	Indoor
5	3.38	1.09	3.81	1.03	Indoor
6	3.25	1.29	3.57	1.16	Indoor
7	3.31	1.25	3.48	1.25	Indoor
8	3.81	1.22	3.29	0.90	Outdoor

Average rating Photographs no. 1–8	3.27	1.19	3.39	1.16	
Average rating – indoor	3.25	1.19	3.60	1.15	
Average rating – outdoor	3.31	1.23	2.79	1.00	


*The scale was from 1 to 5.*

Finally, we calculated correlations between the duration of the walk, walking speeds in specific sections and the rating of the photographs. In the condition priming with trees we found the significant correlation between the duration of the walk and the rating of photographs (*r* = 0.35, *p* < 0.05) and the significant correlation between the duration of the walk and average walking speed (*r* = -0.40, *p* < 0.05) which shows that higher level of liking the photographs with trees resulted in the slower walking speed. Correlations between walking speeds in specific sections of the route and the rating of photographs were significant (except the sections 1 and 2), the highest correlation was found for the group of sections 5, 6, and 7 (*r* = 0.43, *p* < 0.05) with the higher perceived natural character. In the condition priming with forests we did not find any significant correlation. Interestingly, the correlation coefficients showed opposite trend relative to priming with threes. Similarly, in the condition priming with malls we did not find any significant correlation, but the correlation coefficients showed the same trend as in priming with threes.

### Discussion

The results showed that priming with trees had almost an equal effect on walking speed as priming with forests in comparison with priming with malls. Although priming with trees resulted in slightly slower walking speed than priming with forests, this difference was not significant. The participants primed with trees and forests consistently walked more slowly than the participants primed with malls on all sections of the route. Importantly, additional evidence comes from the section located close to the university building. The data revealed that walking speed was equal between the priming with trees and priming with forest conditions and faster under the priming with malls condition. These findings suggest that priming with photographs of both types of vegetation had an immediate effect on walking speed regardless of the characteristics of the walking route.

However, the analysis of correlations between liking of photographs used as the primes and the walking speed provides a contradictory conclusion. Whereas, the level of liking of photographs with trees was significantly associated with slower walking speed, the level of liking of forests was non-significantly associated with faster walking speed consistently in all sections of the route. These results suggest that congruity between the prime and the environmental setting of the walking route might also play some role.

Similar to Experiment 1, the walking speed differed between the groups of sections, where sections 5, 6, and 7 with more natural characteristics were associated with the slowest walking speed.

## General Discussion

We investigated whether priming with photographs of various environmental settings has an effect on the speed of a subsequent walk. The results convincingly showed that priming with photographs of urban greenery and trees, as well as with photographs of forests, resulted in decreased walking speed relative to the other experimental conditions. Contrary to our expectations, we failed to convincingly support the assumption that priming would make the mental representation of certain information (a specific environmental setting) more accessible, which would result in approach behavior associated with a decrease in the walking speed caused by priming congruent with the setting. Conversely, we also failed to find avoidance behavior (increase in the walking speed) caused by priming incongruent with the setting. Although our data might partly support this prediction, the evidence is weak. We found no differences between priming with malls and the no-prime condition in Experiment 1, and differences between priming with trees and forests in Experiment 3 were small and non-significant.

First, the intensity of priming is worth commentary. Our intention was to examine an effect of relatively gentle stimuli, therefore we used photographs presented on sheets of paper as the priming material. To ensure that photographs were carefully perceived by participants, they were asked to rate the level of their liking. However, the drawback of this procedure was that the duration of the priming was not equal in all participants. It has been shown that duration of priming may influence its effect (e.g., Dijksterhuis and van Knippenberg, 1998). However, we assumed that differences in the duration of priming between individual participants in our experiments would not be so large as to greatly influence the behavioral effect. Clearly, future research can also check the potential effect of small differences in the duration of priming and examine the effect of more intense stimuli. Large slides or even videos accompanied by corresponding sounds (e.g., bird sounds, see Ratcliffe et al., 2013) might increase the effect of priming and lead to more salient results. Another point is the purpose of the walk. Our participants knew that they were taking part in an experiment, and moreover, they were equipped with a camera, which may have been disturbing and different from ordinary recreational walks. More realistic conditions could also lead to more salient results.

Importantly, we found that the differences in walking speeds under particular priming conditions appeared in the section located close to the university building. This result suggests that the effect of priming on walking speed may appear immediately after the priming and independently of specific environmental settings. The same behavioral response after priming was observed in the studies by Bargh et al. (1996), Aarts and Dijksterhuis (2002), and Cesario et al. (2006).

There is another possible explanation of the observed effect. The effect of priming with vegetation might also be caused by the positive psychological effect of viewing nature. Evidence shows that viewing nature in the form of photographs, videos or virtual computer-generated nature has a restorative effect that is also manifested in decreased stress levels and increased positive affect (e.g., Ulrich et al., 1991, 2003; Hartig et al., 1999; de Kort et al., 2006; Valtchanov et al., 2010; Brown et al., 2013; Jiang et al., 2014; Felnhofer et al., 2015). However, emotion research has not provided much information about the association between affect and walking speed. Studies have analyzed people’s style of walking, which reflects certain basic emotions, such as happiness, sadness, anger, or pride (e.g., Montepare et al., 1987; De Meijer, 1989; Crane and Gross, 2007; Hicheur et al., 2013). Such research has shown that happiness is associated with faster walking speed (Montepare et al., 1987), whereas gait patterns combined with sadness are characterized by slower walking speed (Michalak et al., 2009). However, there is no evidence that viewing photographs of natural scenes yields as strong and immediate an emotional response as happiness, for example. Kaplan (1984) claimed that exposure to natural settings instills peacefulness and tranquility, where tranquility is defined as a positive affect with low arousal (Russell, 1980). Johansson et al. (2011) observed an increasing feeling of tranquility and decreasing feeling of time pressure after an urban walk in contrast to a street walk. There is also evidence that tranquility correlates with a preference for natural settings (Herzog and Bosley, 1992). Consistent with these considerations, we suggest that priming with photographs of natural settings might elicit the experience of tranquility, which results in a slower walking speed relative to other conditions. This effect may be the reason why participants who were exposed to photographs of urban greenery, trees or forests walked more slowly than participants who were exposed to photographs of malls or not exposed to photographs.

Of course, this explanation is hypothetical and requires more future research. The drawback is that our research paradigm did not enable us to record mood changes directly after priming. Rather, we recorded them when the participants completed their walk. It was not suitable to measure changes in mood immediately after the priming because asking the participants to complete any other questionnaire just after the priming might have disrupted the effect of the priming and have prolonged the interval between the priming and beginning of the walk. As we have seen, in all conditions, negative mood decreased after the walk. The described change in mood, which is in agreement with findings showing that walking in outdoor settings improves mood (Focht, 2009), is most likely a reflection of participants’ reactions to their experiences during the outdoor walk and not to the priming stimuli presented before the walk.

Interestingly, in Experiment 3, the group of female participants primed by malls rated photographs of malls higher than the participants who evaluated photographs of natural scenes. Although there are people who dislike shopping, it is known that some individuals seek hedonic experiences in shopping centers (e.g., Arnold and Reynolds, 2003). Gender differences in shopping preferences have already been mentioned. Recently, Staats et al. (2016) showed that an outdoor park setting had the highest restorative potential and was most liked, but the results showed that malls also had some restorative potential, particularly for women in their sample. Thus, viewing photographs of well-designed shopping malls may also elicit positive emotions.

Another issue that requires comment is the durability of the effect of priming. In previous priming studies (Bargh et al., 1996; Aarts and Dijksterhuis, 2002; Cesario et al., 2006), participants walked several meters after completing the priming task. In our experiments, they walked a route ranging from 1.2 to 1.9 km. In all sections of the routes, the effect of particular types of primes was almost equal. Furthermore, while the participants were primed directly before the walk in Experiment 3, in Experiment 1 and Experiment 2, the interval between the priming and the walk was as long as 5–6 min. Regardless of the period between priming and the beginning of the walk, the effect of priming was almost equal. It seems that time between priming and response may not be an important factor, unless it is not filled by a concurrent or disturbing stimuli or activity. After all, in typical priming experiments within the field of social psychology, the task after presentation of a prime lasts some time (approximately 10–20 min) without any noticed decay of the priming effect. Moreover, there are also known experiments from social psychology where a priming effect was noticed even eight months after the exposure to the prime (Carter et al., 2011).

To conclude, we did observe that priming with photographs with vegetation caused a decrease in overall walking speed with a medium effect size relative to other experimental conditions in all experiments. We provided two possible explanations for the observed behavior. The first explanation would suggest that priming congruent with the environment of the walking route could result in approach behavior associated with slower walking speed. The second was based on the positive psychological effects of viewing nature. We suggest that priming with photographs of natural settings might elicit the experience of tranquility, which results in slower walking speed relative to other conditions. These two explanations would not be in contradiction, as they might be both partly responsible for the observed behavior. Clearly, future research is needed to fully explain the underlying mechanisms.

What are the implications of these findings? First, our study adds further evidence for a broad range of diverse positive effects of viewing natural scenes, ranging from the cognitive benefits of interacting with nature (Berman et al., 2008) to overall improvements in health and well-being (e.g., Velarde et al., 2007) or even greater connectedness to nature (Mayer et al., 2009). We demonstrated the effect of surrogate nature in the form of photographs and showed that exposure to photographs of natural scenes led to decreased walking speed in an urban environment. It is notable that as early as the 1970s, Milgram (1970) described how the effect of crowding, stress, and cognitive overload in large cities was associated with the fast pace of life. It was shown that a fast urban walking speed is associated with a faster pace of life (e.g., Bornstein and Bornstein, 1976; Levine et al., 1989; Levine and Norenzayan, 1999). Moreover, our study demonstrated the effect of viewing natural scenes while walking along a city route. People walking across areas with trees and greenery walked slower relative to locations without greenery.

Second, our findings also enhance our knowledge of the diverse mechanisms of priming in psychology. Importantly, in this experiment, new methods of indirect priming and measuring behavioral responses (walking in a natural setting) were used. Our study may thus also contribute to the debate about priming in social psychology (e.g., Bargh, 2006; Custers and Aarts, 2010), especially because several recent studies (Doyen et al., 2012; Harris et al., 2013; LeBel and Campbell, 2013) have failed to replicate the results of older priming studies.

The study has some limitations. First, precise experimental control would require us to also conduct experiments in environments related to other primes. However, doing so is almost impossible for practical reasons. It would be difficult to implement such conditions not least because of the many uncontrolled external variables in shopping malls and the physical distance of submontane forests. Importantly, the current experiments used a novel research paradigm. Clearly, many combinations of conditions and stimuli are possible, but they cannot be used within one study. Future research would require a more precise assessment of mood changes to strengthen our claim about the role of mood in affecting walking speed. It is difficult to objectively measure cognitive engagement and involvement in an environment. In future research, eye tracking methods could be used to determine whether a specific prime can influence interest in viewing particular objects in an environment. Further, it would be useful to measure walking speed directly after priming at the very beginning of the walk. Such research could further strengthen our assumption about the immediate response to viewing photographs of natural scenes. The use of a student sample is also a limitation of our study. On the other hand, compared with a representative sample from the general population, our student sample comprising young people from the same faculty is in many respects quite consistent. In this way, we could suppress the influence of diverse external variables.

## Author Contributions

MF designed the experiments, analyzed the data, and wrote the article. LR acquired and analyzed the data and contributed to writing the article.

## Conflict of Interest Statement

The authors declare that the research was conducted in the absence of any commercial or financial relationships that could be construed as a potential conflict of interest.
