# Supplementary material for: The Effect of Priming with Photographs of Environmental Settings on Walking Speed in an Outdoor Environment
Source: Front Psychol. 2017 Jan 26;8:73. doi: 10.3389/fpsyg.2017.00073 (PMC5266685; doi:10.3389/fpsyg.2017.00073)

The photographs used as the priming material

The photographs of trees 1 - 4

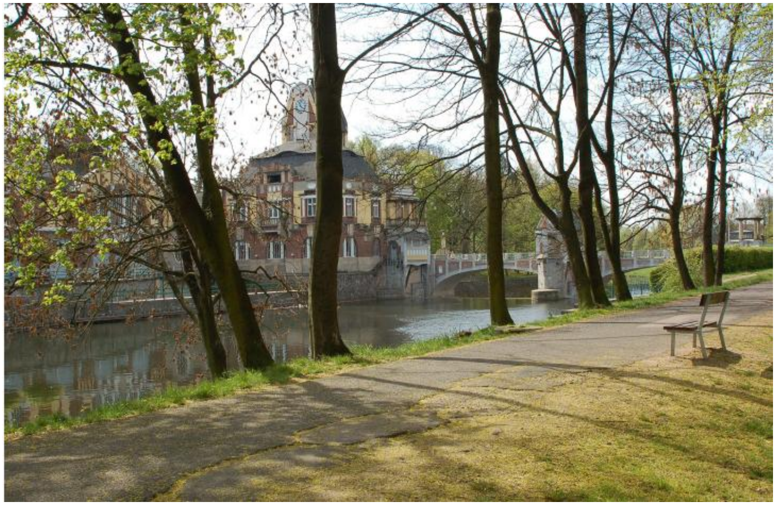

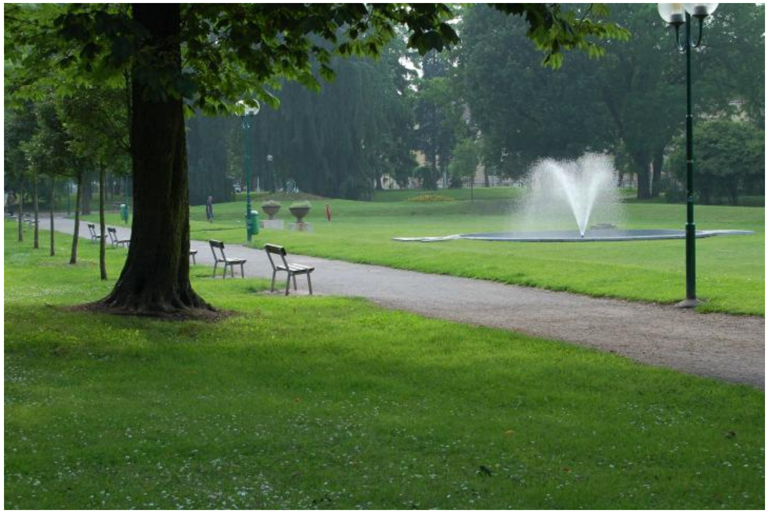

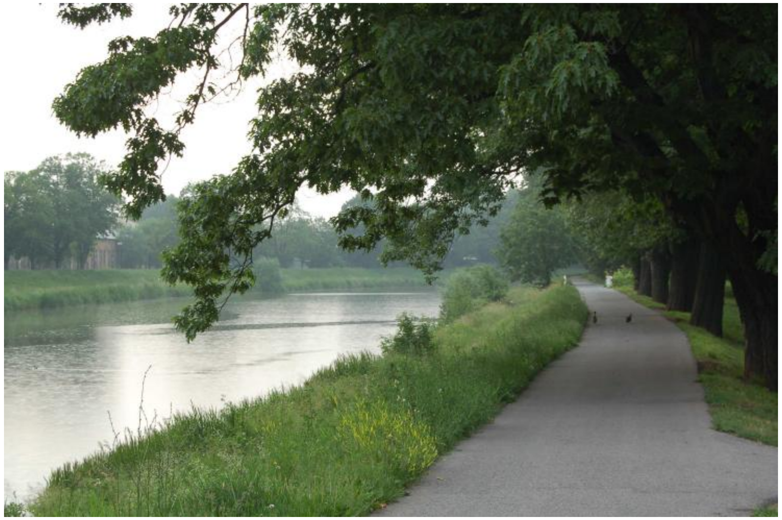

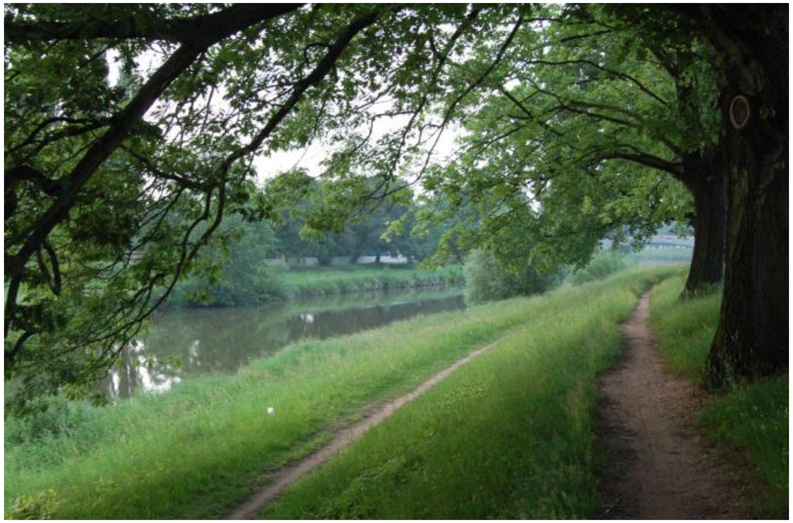

The photographs of trees 5 - 8

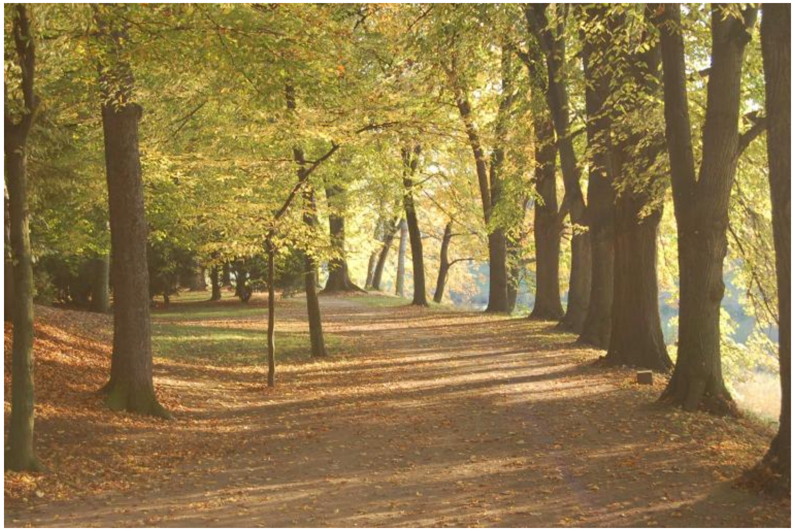

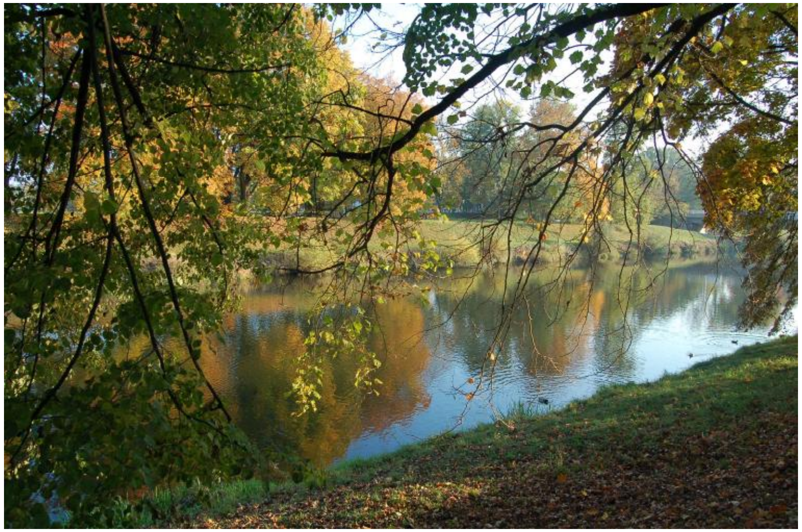

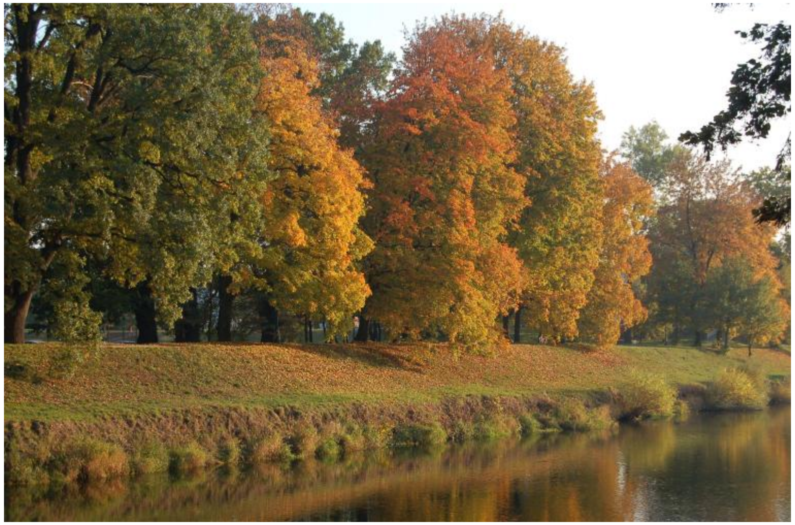

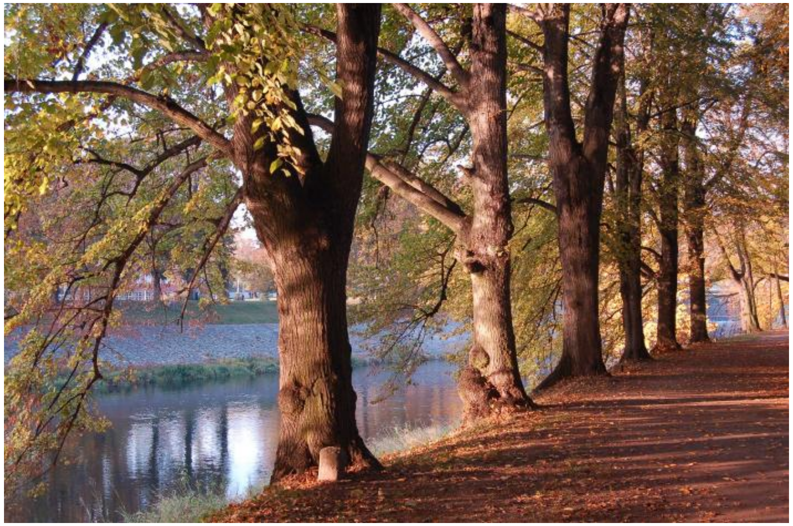

The photographs of malls 1 - 4

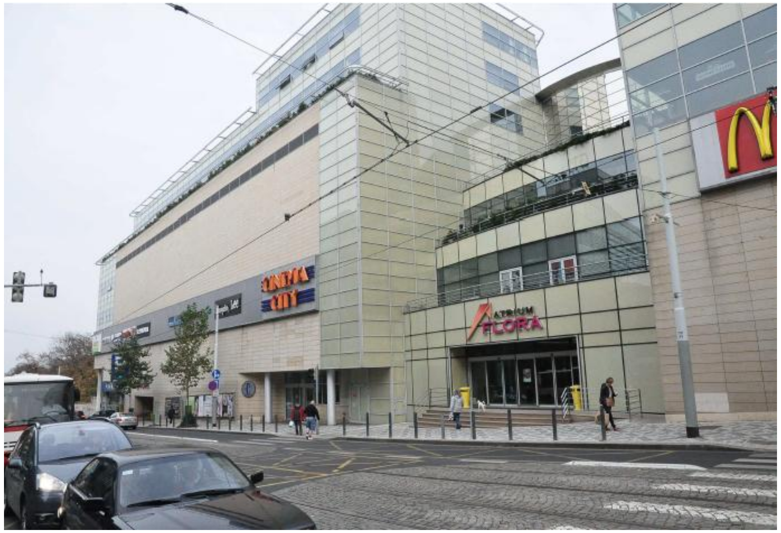

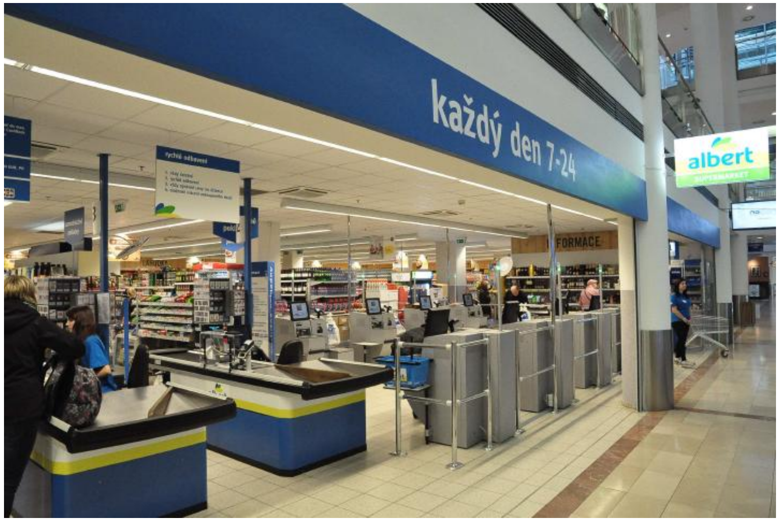

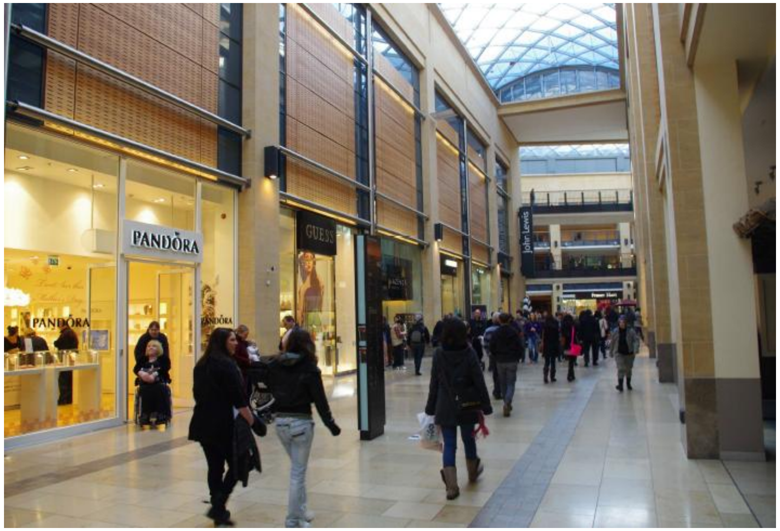

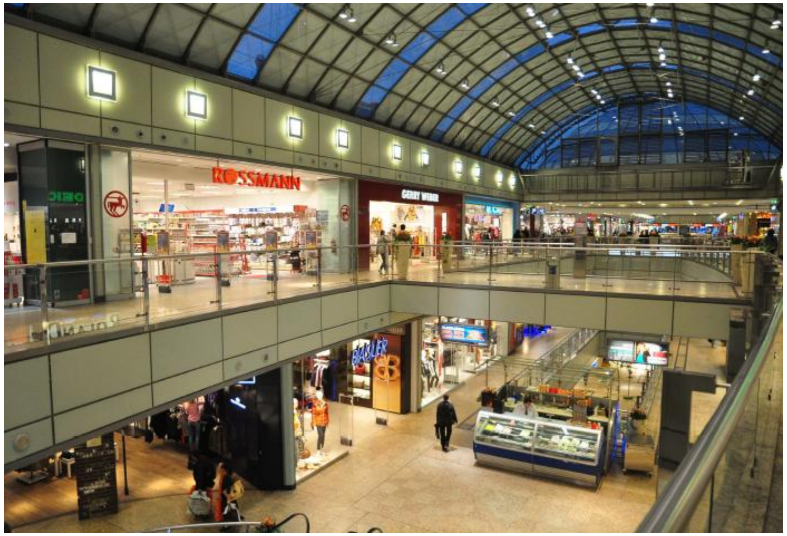

The photographs of malls 5 - 8

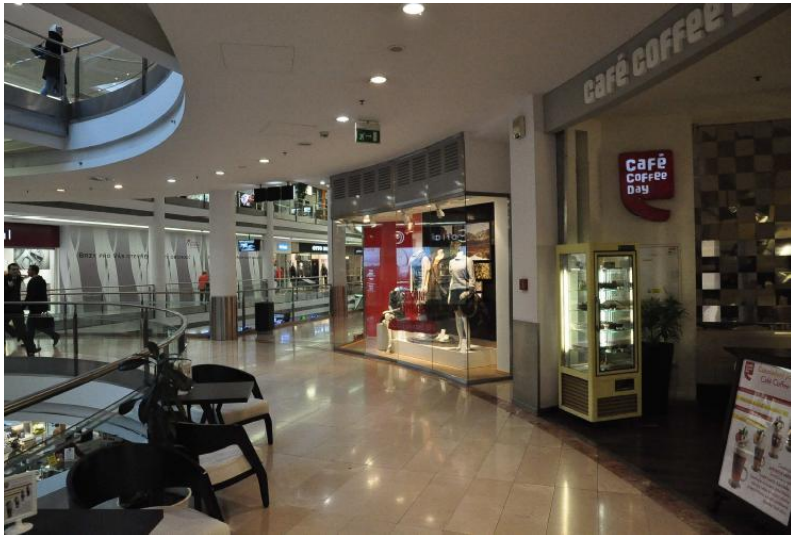

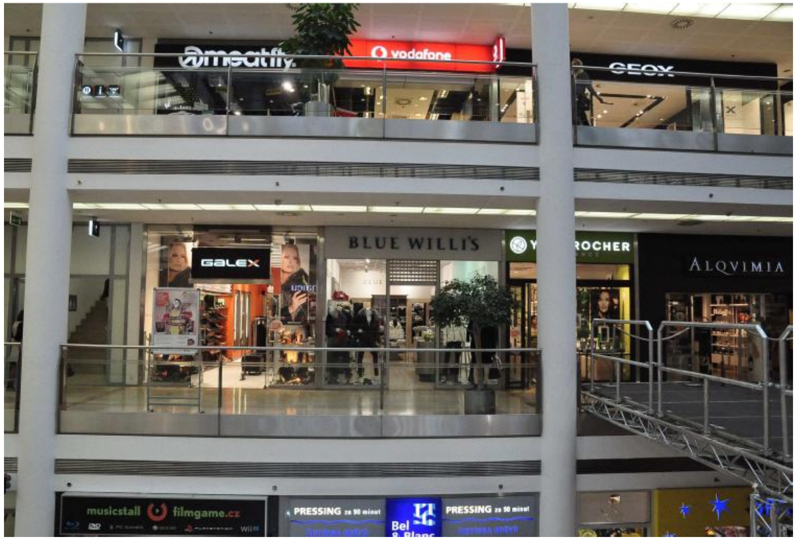

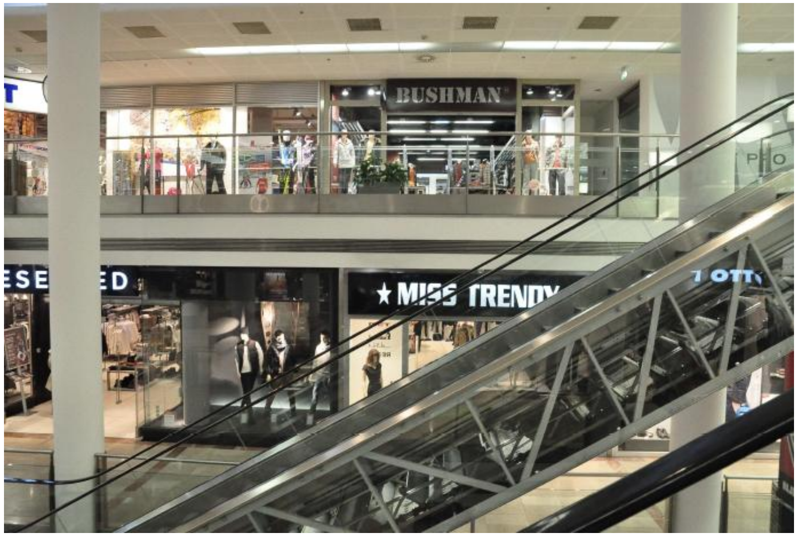

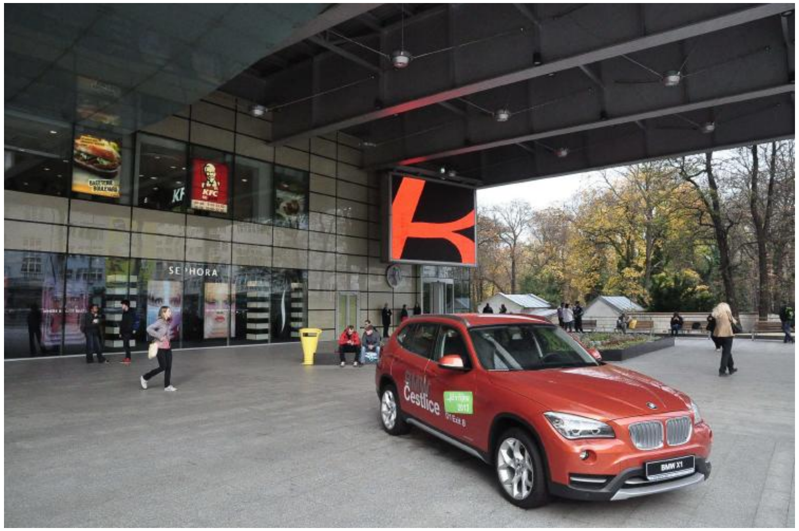

The photographs of forests 1 - 4

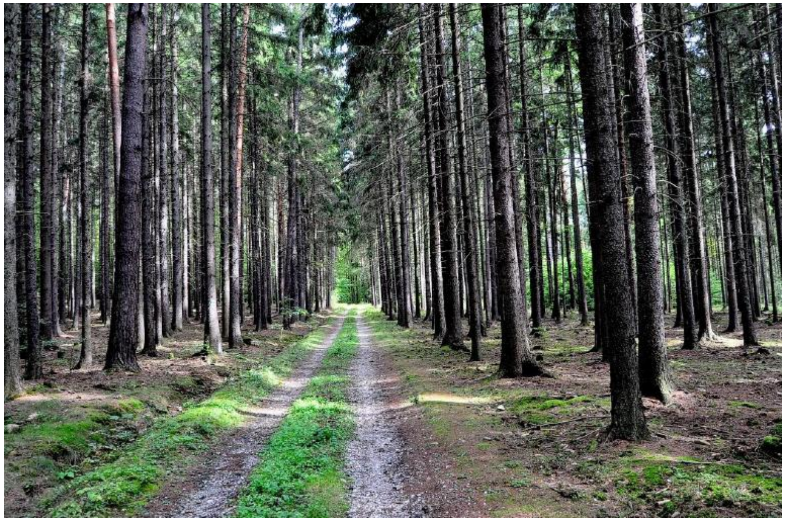

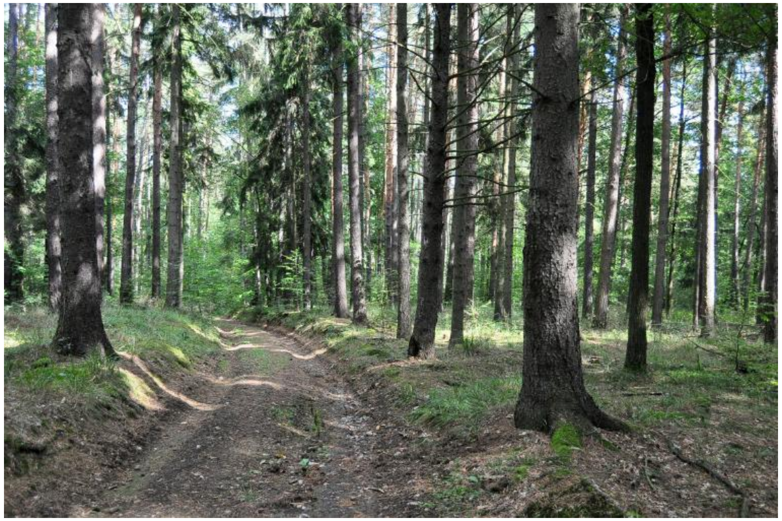

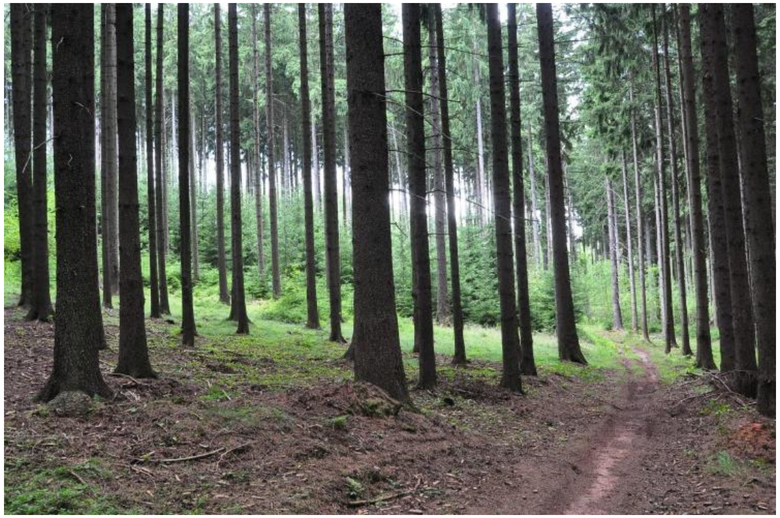

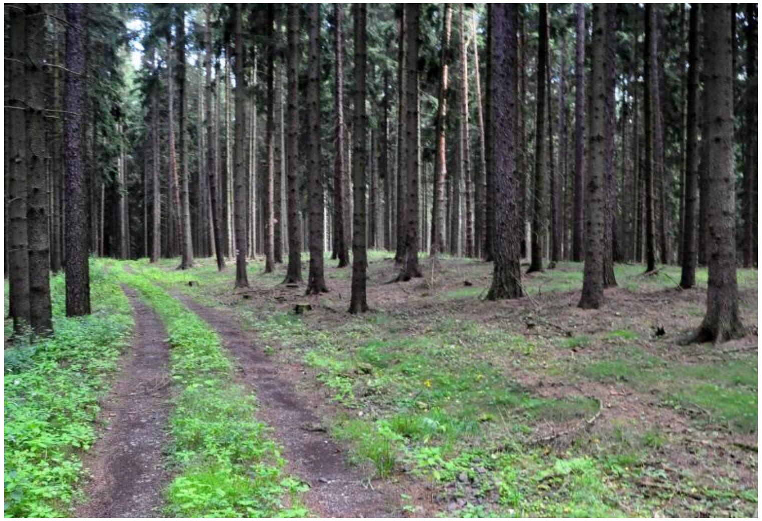

The photographs of forests 5 - 8

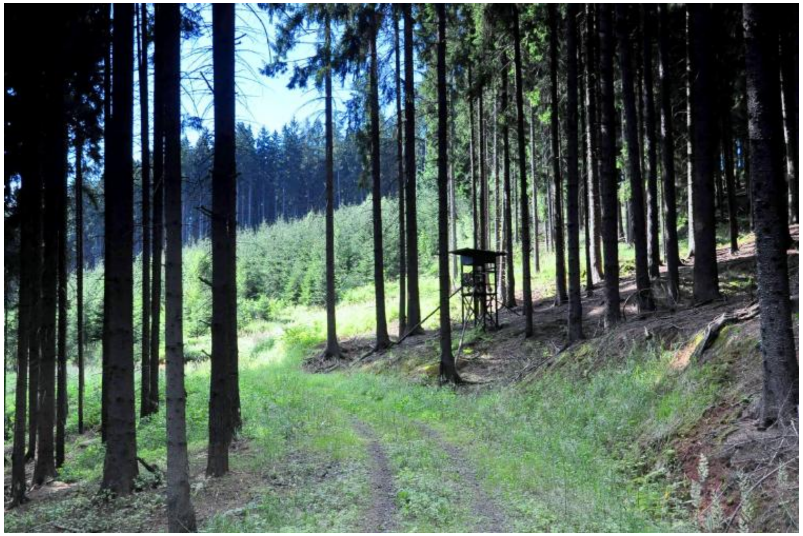

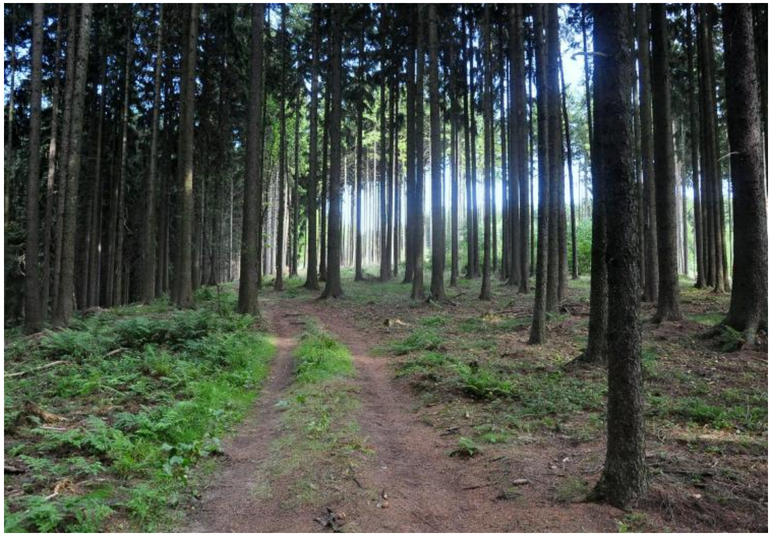

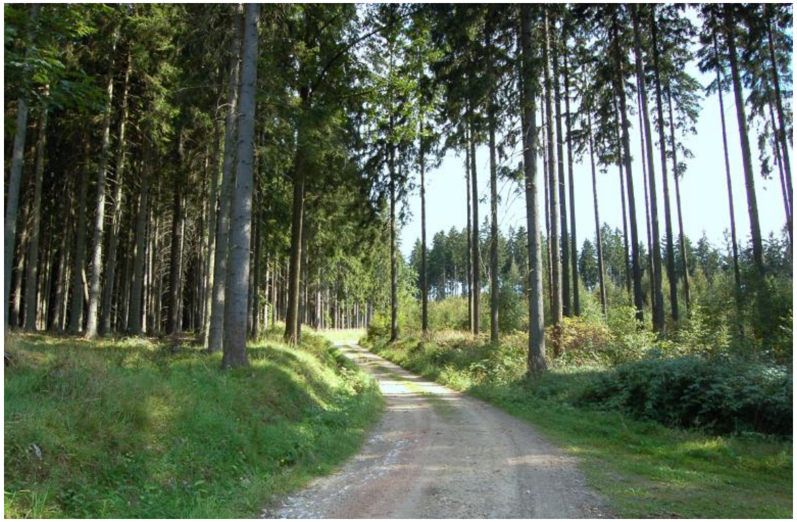

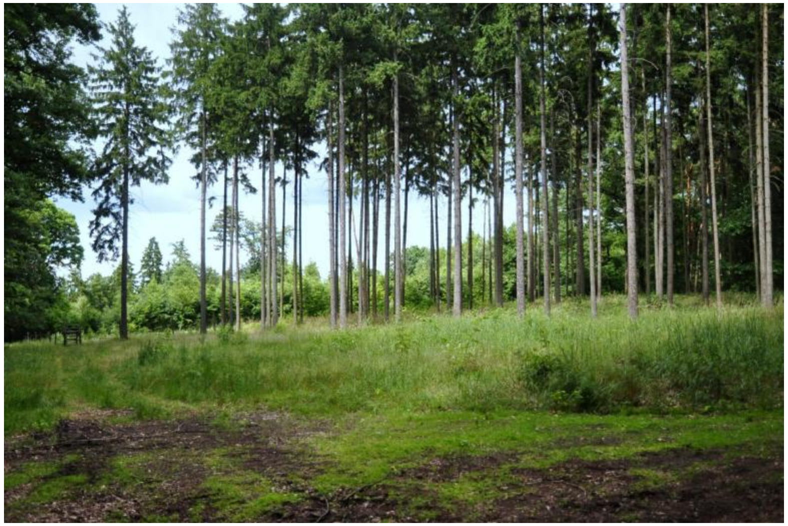

Supplement: Supplementary file 1 [file Data_Sheet_1.PDF]
